# Clarithromycin prevents preterm birth and neonatal mortality by dampening alarmin-induced maternal–fetal inflammation in mice

**DOI:** 10.1186/s12884-022-04764-2

**Published:** 2022-06-20

**Authors:** Jose Galaz, Roberto Romero, Marcia Arenas-Hernandez, Marcelo Farias-Jofre, Kenichiro Motomura, Zhenjie Liu, Naoki Kawahara, Catherine Demery-Poulos, Tzu Ning Liu, Justin Padron, Bogdan Panaitescu, Nardhy Gomez-Lopez

**Affiliations:** 1 Perinatology Research Branch, Division of Obstetrics and Maternal-Fetal Medicine, Division of Intramural Research, Eunice Kennedy Shriver National Institute of Child Health and Human Development, National Institutes of Health, U.S. Department of Health and Human Services (NICHD/NIH/DHHS), Bethesda, MD, 20892, and Detroit, MI 48201 USA; 2 Department of Obstetrics and Gynecology, Wayne State University School of Medicine, Detroit, MI 48201 USA; 3 Division of Obstetrics and Gynecology, School of Medicine, Faculty of Medicine, Pontificia Universidad Catolica de Chile, 8330024 Santiago, Chile; 4 Department of Obstetrics and Gynecology, University of Michigan, Ann Arbor, MI 48109 USA; 5 Department of Epidemiology and Biostatistics, Michigan State University, East Lansing, MI 48824 USA; 6 Center for Molecular Medicine and Genetics, Wayne State University, Detroit, MI 48201 USA; 7 Detroit Medical Center, Detroit, MI 48201 USA; 8 Department of Biochemistry, Microbiology and Immunology, Wayne State University School of Medicine, Detroit, MI 48201 USA

**Keywords:** Amniotic cavity, Antibiotic, Cytokine, Gene expression, HMGB1, Macrolide, Sterile intra-amniotic inflammation

## Abstract

**Background:**

One of every four preterm neonates is born to a woman with sterile intra-amniotic inflammation (inflammatory process induced by alarmins); yet, this clinical condition still lacks treatment. Herein, we utilized an established murine model of sterile intra-amniotic inflammation induced by the alarmin high-mobility group box-1 (HMGB1) to evaluate whether treatment with clarithromycin prevents preterm birth and adverse neonatal outcomes by dampening maternal and fetal inflammatory responses.

**Methods:**

Pregnant mice were intra-amniotically injected with HMGB1 under ultrasound guidance and treated with clarithromycin or vehicle control, and pregnancy and neonatal outcomes were recorded (*n* = 15 dams each). Additionally, amniotic fluid, placenta, uterine decidua, cervix, and fetal tissues were collected prior to preterm birth for determination of the inflammatory status (*n* = 7–8 dams each).

**Results:**

Clarithromycin extended the gestational length, reduced the rate of preterm birth, and improved neonatal mortality induced by HMGB1. Clarithromycin prevented preterm birth by interfering with the common cascade of parturition as evidenced by dysregulated expression of contractility-associated proteins and inflammatory mediators in the intra-uterine tissues. Notably, clarithromycin improved neonatal survival by dampening inflammation in the placenta as well as in the fetal lung, intestine, liver, and spleen.

**Conclusions:**

Clarithromycin prevents preterm birth and improves neonatal survival in an animal model of sterile intra-amniotic inflammation, demonstrating the potential utility of this macrolide for treating women with this clinical condition, which currently lacks a therapeutic intervention.

**Supplementary Information:**

The online version contains supplementary material available at 10.1186/s12884-022-04764-2.

## Background

Preterm birth is the leading cause of neonatal morbidity and mortality worldwide [1–3], with two-thirds of all cases preceded by spontaneous preterm labor [4]. The latter is a syndrome comprising multiple etiologies [4, 5], with intra-amniotic infection and/or inflammation as the only well-established causal link to preterm birth [6–21]. Intra-amniotic inflammation was thought to be exclusively initiated by the invasion of microbes into the amniotic cavity (i.e., intra-amniotic infection) [22–25]. Yet, the advancement of molecular microbiological techniques has allowed for the discovery of a new entity, sterile intra-amniotic inflammation, in which elevated concentrations of cytokines [i.e., interleukin (IL)-6] occur in the absence of detectable microorganisms [26–30]. From an immunological perspective, sterile inflammation is triggered by danger signals or damage-associated molecular patterns (DAMPs; also known as alarmins) released upon cellular stress, senescence, or necrosis [31–34]. Therefore, we have proposed that elevated concentrations of alarmins in the amniotic cavity are responsible for activating the inflammatory cascade leading to preterm labor and birth [27, 35]. In support of this concept, the concentrations of several classical alarmins, namely high-mobility group box-1 (HMGB1) [27, 36], S100 calcium-binding protein-B (S100B) [37], IL-1α [38, 39], and heat-shock protein 70 (HSP70) [40], are increased in women with intra-amniotic inflammation. Notably, sterile intra-amniotic inflammation is more prevalent than intra-amniotic infection in women with preterm labor and intact membranes [27]. In addition, patients with both sterile intra-amniotic inflammation and an increased amniotic fluid concentration of HMGB1 deliver sooner than those with a lower concentration of this alarmin [27], indicating that HMGB1 can serve as a predictor of preterm delivery. Indeed, we have mechanistically demonstrated that elevated concentrations of HMGB1 [10, 17], as well as other alarmins [14, 16, 21, 41], in the amniotic cavity induce sterile inflammation and cause preterm labor and birth. Therefore, we are actively engaged in finding strategies to prevent preterm labor and birth by inhibiting sterile intra-amniotic inflammation.

The mechanisms that lead to sterile intra-amniotic inflammation involve the activation of inflammatory pathways such as the NLR family pyrin domain-containing-3 (NLRP3) inflammasome [14, 16, 21, 42–44]. Hence, we have proposed the use of inhibitors of NLRP3 inflammasome activation to treat sterile intra-amniotic inflammation and to prevent preterm labor and birth [14]. However, a limitation of this approach is that a few cases of spontaneous preterm labor categorized as sterile intra-amniotic inflammation may be associated with undetectable microorganisms, thus the blockade of the NLRP3 inflammasome could limit the host response mechanisms required for clearance of such a pathogen. Alternatively, we have recently shown that treatment with betamethasone, a widely used corticosteroid, prevents preterm birth; nevertheless, such an approach did not rescue neonatal mortality induced by the intra-amniotic administration of HMGB1 [17]. Therefore, a strategy that not only prevents preterm birth but also improves neonatal survival by dampening the intra-amniotic inflammatory response induced by alarmins is urgently needed.

Recent studies have shown that specific antibiotics, such as clarithromycin, not only display effective anti-microbial properties in women with intra-amniotic infection but also exert anti-inflammatory effects in the amniotic cavity [45–49]. Therefore, we propose that clarithromycin, which is already approved for clinical use in pregnant women, could represent a viable treatment for women presenting with sterile intra-amniotic inflammation and risk of preterm delivery. Importantly, clarithromycin is the macrolide that most efficiently crosses the placenta [50, 51]. Indeed, a recent clinical investigation showed a reduction in the severity of the intra-amniotic inflammatory response (as indicated by amniotic fluid IL-6 levels) in women who received treatment with clarithromycin [48]. However, to date, there has been no mechanistic demonstration showing that treatment with clarithromycin prevents preterm birth and adverse neonatal outcomes. Furthermore, the anti-inflammatory effects exerted by clarithromycin in the maternal–fetal tissues have not been investigated.

In the current study, we utilized an established model of sterile intra-amniotic inflammation induced by the alarmin HMGB1 to evaluate whether treatment with clarithromycin prevents preterm birth and adverse neonatal outcomes. Moreover, we investigated the maternal and fetal inflammatory responses in mice treated with clarithromycin to elucidate the anti-inflammatory effects of this macrolide in the setting of sterile intra-amniotic inflammation.

## Methods

### Mice

C57BL/6 mice were purchased from The Jackson Laboratory (Bar Harbor, ME, USA) and bred in the animal care facility at the C.S. Mott Center for Human Growth and Development at Wayne State University (Detroit, MI, USA), according to protocols previously established by our group [12–17, 52–55]. Briefly, mice were housed with a 12 h light:12 h dark cycle (lights on from 6 am – 6 pm). Females of 8–12 weeks old were bred with males of proven fertility and checked daily between 8:00–9:00 a.m. for the appearance of a vaginal plug, which was considered as 0.5 day *post coitum* (dpc). After observing a vaginal plug, females were separated from the males and monitored daily, with a weight gain of at least 2 g by 12.5 dpc indicating pregnancy. All procedures were approved by the Institutional Animal Care and Use Committee (IACUC) at Wayne State University (Protocol No. 18–03-0584 and 21–04-3506). All animals were randomly assigned to experimental or control groups prior to the following experiments. Due to randomization, investigators were not blinded to group assignment.

### Intra-amniotic administration of HMGB1

**Fig. 1:**
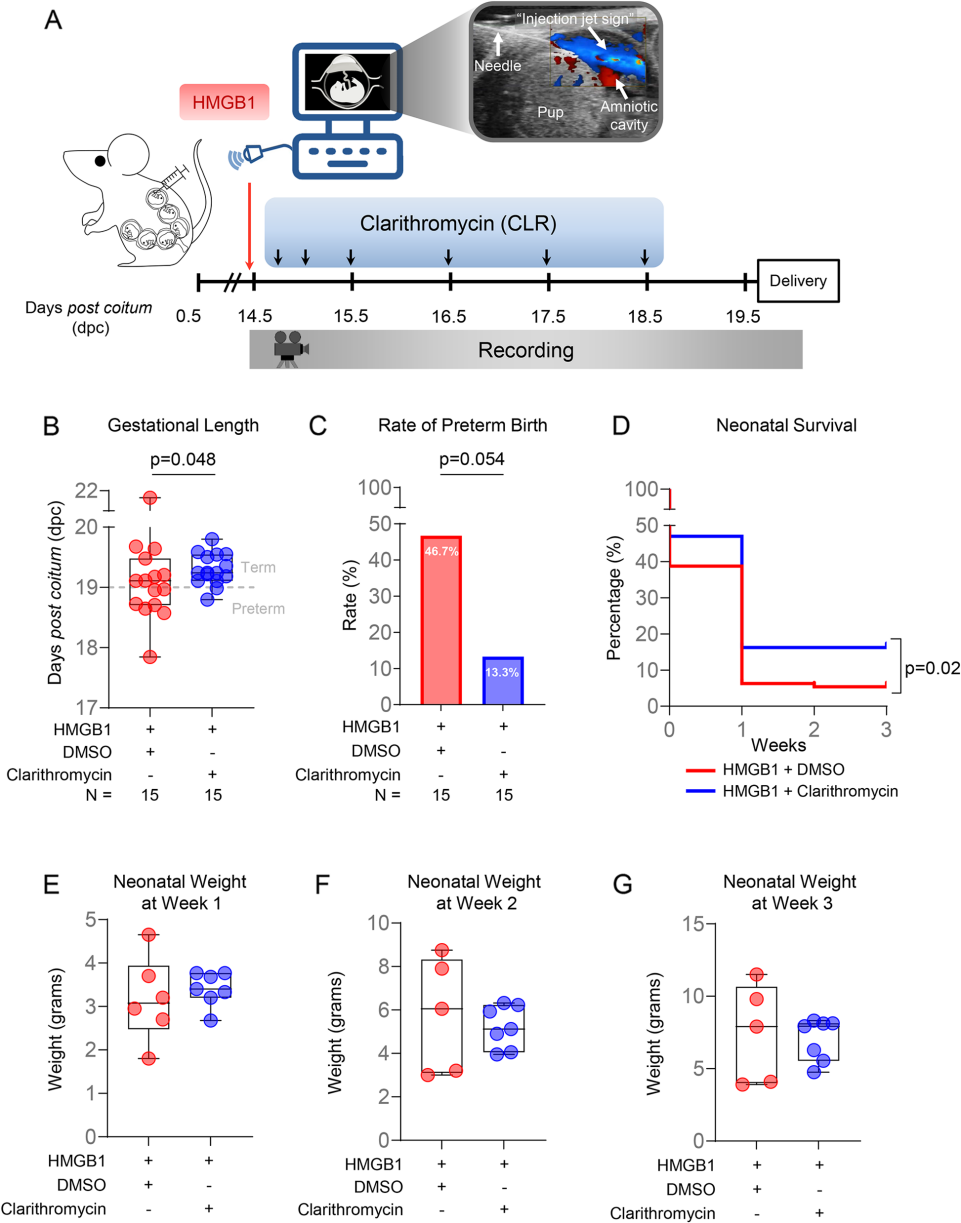
Clarithromycin prevents preterm birth and improves neonatal survival induced by intra-amniotic injection of HMGB1. **A** Dams were intra-amniotically injected with HMGB1 in each sac under ultrasound guidance on 14.5 days *post coitum* (dpc) and treated with 75 mg/kg of clarithromycin (CLR; *n* = 15) or DMSO (vehicle control; *n *= 15) at 6, 12, 24, 48, 72, and 96 h post-injection. Dams were monitored until delivery, and neonates were followed until three weeks of age. **B** Gestational length (dpc) of dams intra-amniotically injected with HMGB1 and treated with CLR (blue dots) or DMSO (red dots). Data are represented as box-and-whisker plots with medians, interquartile ranges, and min/max ranges. *P*-value was determined using the one-sided Mann–Whitney U-test. **C** Rate of preterm birth of dams intra-amniotically injected with HMGB1 and treated with CLR (blue bar plot) or DMSO (red bar plot). *P*-value was determined using the one-sided Fisher’s exact test. **D** Kaplan–Meier survival curve showing the percentage of surviving neonates at 1, 2, and 3 weeks of age from dams intra-amniotically injected with HMGB1 and treated with CLR (blue line) or DMSO (red line). *P-*value was determined using the Mantel-Cox test. **E–G** Neonatal weights per litter at 1, 2, and 3 weeks of age from dams intra-amniotically injected with HMGB1 and treated with CLR [blue dots, 2–6 (mean = 3.8) neonates/litter] or DMSO [red dots, 1–2 (mean = 1.5) neonates/dam]. Data are represented as box-and-whisker plots with medians, interquartile ranges, and min/max ranges. *P*-value was determined using the one-sided Mann–Whitney U-test

The intra-amniotic administration of HMGB1 was performed as previously described [10, 17]. Briefly, dams were anesthetized on the morning of 14.5 dpc (8:00 – 10:00 am) by inhalation of 2% isoflurane [Fluriso™ (Isoflurane, USP) Vetone Boise, ID, USA] and 1–2 L/min of oxygen in an induction chamber, and a mixture of 1.5–2% isoflurane and 1.5–2 L/min of oxygen was used to maintain anesthesia. Mice were positioned on a heating pad and stabilized with adhesive tape, and fur was removed from the abdomen and thorax using Nair cream (Church & Dwight Co., Inc., Ewing, NJ, USA). Body temperature was detected with a rectal probe (VisualSonics, Toronto, ON, Canada) throughout the procedure, and respiratory and heart rates were monitored by electrodes embedded in the heating pad. An ultrasound probe was fixed and mobilized with a mechanical holder, and the transducer was slowly moved toward the abdomen [56]. The ultrasound-guided intra-amniotic injection of recombinant human HMGB1 (*n* = 30; Biolegend, San Diego, CA, USA) at a concentration of 9 ng dissolved in 100 μL of sterile 1X phosphate-buffered saline (PBS; Life Technologies, Grand Island, NY, USA) was performed in each amniotic sac using a 30G needle (BD PrecisionGlide Needle; Becton Dickinson, Franklin Lakes, NJ, USA) [10]. This dose of HMGB1 was determined from the pathophysiological amniotic fluid concentrations found in women with sterile intra-amniotic inflammation [27]. Successful intra-amniotic injection was verified by using color Doppler ultrasound to identify the “injection jet sign” [17] (Fig. 1A). After ultrasound completion, mice were placed under a heat lamp for recovery, which was defined as when the mouse resumed normal activities, such as walking and responding, and typically occurred within 10 min after removal from anesthesia. After recovery, dams were monitored via video camera to observe pregnancy and neonatal outcomes.

### Clarithromycin treatment of mice intra-amniotically injected with HMGB1

After the ultrasound-guided intra-amniotic injection of HMGB1, dams were randomized to receive subcutaneous treatment with either 75 mg/kg of clarithromycin (*n* = 15; Sigma-Aldrich, St. Louis, MO, USA) or its vehicle control [*n* = 15; diluted dimethyl sulfoxide (DMSO), Sigma-Aldrich] at 6, 12, 24, 48, 72, and 96 h post-intra-amniotic injection. Clarithromycin (reconstituted with DMSO) and DMSO (vehicle control) were diluted in sterile 5% dextrose water. Dams were continuously observed between each treatment via video camera to evaluate pregnancy and neonatal outcomes. The dose of clarithromycin was determined based on a previous study [57], suggesting that a 75 mg/kg/day dose of clarithromycin in mice was equivalent to human oral clarithromycin dosages (ranging from 250 mg twice daily to 500 mg twice daily in adults [58]). The latter dose has been demonstrated to ameliorate intra-amniotic infection or inflammation in a subset of women with preterm labor and intact membranes [47], cervical insufficiency [46], or preterm prelabor rupture of the membranes [45] when it is administered together with other antibiotics (ceftriaxone and metronidazole) or alone [48]. Moreover, *Ureaplasma parvum*-induced preterm birth and adverse neonatal outcomes were shown to improve after treatment with the same dose of clarithromycin in mice [15].

### Video monitoring of pregnancy and neonatal outcomes

Experimental mice were continuously monitored with a video camera (Sony Corporation, Tokyo, Japan) to record pregnancy outcomes, using observational protocols established by our group [12, 14–17, 52–55]. Primary measured outcomes included the rates of preterm birth and neonatal mortality. Gestational length was calculated for each dam as the elapsed time from the presence of the vaginal plug until the appearance of the first pup in the cage bedding. Preterm birth, defined as delivery occurring before 19.0 dpc, was calculated as the proportion of females delivering prior to 19.0 dpc among the total number of mice per study group [17]. Late preterm birth was defined as delivery occurring between 18.0 and 19.0 dpc [17]. The rate of neonatal mortality was calculated for each litter as the proportion of delivered pups found dead among the total litter size. A total of 45 neonates born to control dams and 55 neonates born to dams treated with clarithromycin were observed daily until three weeks postpartum to evaluate neonatal weight and survival. Neonates found dead within the first week of life were excluded from comparisons of neonatal weight per litter.

### Tissue sampling from dams intra-amniotically injected with HMGB1

Pregnant mice were intra-amniotically injected with HMGB1 under ultrasound guidance on 14.5 dpc. Dams were then treated with either 75 mg/kg of clarithromycin (*n* = 7) or DMSO (*n* = 8) at 6, 12, 24, 48, 72, and 96 h post-intra-amniotic injection as described above. On 18.5 dpc, two hours after the last dose of clarithromycin or DMSO (i.e., 98 h post-HMGB1 injection), mice were euthanized by exsanguination (under anesthesia), and tissue collection was performed to obtain the decidua, uterus, cervix, fetal membranes, placenta, fetal lung, fetal intestine, fetal liver, and fetal spleen. Briefly, tissues were either snap-frozen in liquid nitrogen and stored at -80 °C or submerged in RNA*later* solution (Invitrogen/Thermo Fisher Scientific, Baltics UAB, Vilnius, Lithuania). Amniotic fluid was collected from each amniotic sac using a 26G needle. Amniotic fluid samples were centrifuged at 1,300 × g for 10 min at 4 °C, after which the supernatants were separated and kept at -20 °C until analysis.

### RNA isolation, cDNA synthesis, and reverse transcription-quantitative PCR analysis of murine tissues

Total RNA was isolated from the decidua, uterus, cervix, fetal membranes, placenta, fetal lung, fetal intestine, fetal liver, and fetal spleen using QIAshredders, RNase-free DNase sets, and RNeasy Mini kits (all from Qiagen, Hilden, Germany), according to the manufacturer’s instructions, as previously described [15]. The concentrations and integrity of RNA were determined using the Bioanalyzer 2100 (Agilent Technologies, Wilmington, DE, USA). Complementary (c)DNA was synthesized by using SuperScript IV VILO Master Mix (Invitrogen/Thermo Fisher Scientific). Gene expression profiling was carried out on the BioMark system (Fluidigm, San Francisco, CA, USA) with TaqMan gene expression assays (Applied Biosystems/Life Technologies Corporation, Pleasanton, CA, USA) listed in Additional file 2 and Table S1. The expression of multiple reference genes (*Gusb*, *Hsp90ab1*, *Gapdh*, and *Actb*) was averaged within each sample to determine the negative delta threshold cycle (-∆C_T_) values.

### Determination of cytokine concentrations in amniotic fluid

Amniotic fluid samples were assessed for cytokine/chemokine concentrations using the ProcartaPlex mouse cytokine and chemokine panel 1A 36-plex (Invitrogen/Thermo Fisher Scientific), according to the manufacturer’s instructions. For this study, we only report the amniotic fluid concentrations of IL-6, IL-1β, TNF, IL-1α, IL-10, IFNγ, M-CSF, CCL2 (MCP-1), CCL4 (MIP-1α), CCL5 (RANTES), CXCL1 (GRO-α), and CXCL10 (IP-10), as they have been shown to be involved in intra-amniotic inflammation [39]. The rest of the cytokines included in the assay did not differ between study groups, thus were not shown. Plates were read by using the Luminex FLEXMAP 3D (Luminex, Austin, TX, USA) and analyte concentrations were calculated with the Xponent version 4.2 (Luminex). The sensitivities of the assays were as follows: 0.21 pg/mL (IL-6), 0.14 pg/mL (IL-1β), 0.39 pg/mL (TNF), 0.32 pg/mL (IL-1α), 0.69 pg/mL (IL-10), 0.09 pg/mL (IFNγ), 0.02 pg/mL (M-CSF), 3.43 pg/mL (CCL2/MCP-1), 1.16 pg/mL (CCL4/MIP-1β), 0.35 pg/mL (CCL5/RANTES), 0.05 pg/mL (CXCL1/GRO-α), and 0.26 pg/mL (CXCL10/IP-10).

### Statistical analysis

Statistical analyses were conducted using GraphPad Prism version 8.0.1 for Windows (GraphPad Software, San Diego, California, USA, www.graphpad.com), as previously described [17]. A one-sided Fisher’s exact test was used to compare the rates of preterm birth, and a one-sided Mann–Whitney *U*-test was used to compare gestational length, neonatal mortality, neonatal weight, amniotic fluid cytokine/chemokine concentrations, and gene expression between study groups. Kaplan–Meier survival curves were used to plot and compare neonatal survival using the Mantel–Cox test. A *p*-value ≤ 0.05 was considered statistically significant.

## Results

### Treatment with clarithromycin prevents intra-amniotic HMGB1-induced preterm birth and neonatal mortality

We have previously demonstrated a causal relationship between the intra-amniotic injection of HMGB1 and preterm birth in mice [10, 17]. Indeed, we reported that 40% of dams that received intra-amniotic HMGB1 underwent late preterm delivery, resembling the clinical setting in which most preterm deliveries occur after 34 weeks of gestation (i.e., late preterm) [2, 5]. Clarithromycin has emerged as a potential treatment for women presenting with intra-amniotic inflammation and preterm labor [47]; therefore, we first evaluated whether treatment with clarithromycin improves adverse pregnancy and neonatal outcomes in the context of sterile intra-amniotic inflammation induced by HMGB1 (Fig. 1A). The gestational length of dams that received HMGB1 was extended by treatment with clarithromycin compared to that of dams that received HMGB1 and were treated with vehicle control (Fig. 1B). Importantly, 6/7 of the control mice underwent late preterm birth, while only 1/7 underwent early preterm birth (Fig. 1B), which is consistent with our previous report [17]. The extension of the gestational length was reflected by the rate of preterm birth, which was reduced by 33% [from 46.7% (7/15) to 13.3% (2/15)] after treatment with clarithromycin (Fig. 1C). Thus, clarithromycin can ameliorate HMGB1-induced preterm labor and birth.

A major consequence of prematurity is the increased risk of neonatal morbidity and mortality [1, 2, 59], a risk that is exacerbated in the context of intra-amniotic inflammation [24, 60–64]. Therefore, we next evaluated the outcomes of neonates born to dams that received intra-amniotic HMGB1 and were treated with clarithromycin. A greater proportion of neonates born to dams that received HMGB1 and clarithromycin survived up to three weeks of age compared to those born to control dams (Fig. 1D). No differences in litter weight at one (Fig. 1E), two (Fig. 1F), or three (Fig. 1G) weeks of age were found between neonates born to dams that received an intra-amniotic injection of HMGB1 with clarithromycin treatment and those that received vehicle control.

Together, these results demonstrate that clarithromycin can prevent preterm birth and, more importantly, improve neonatal survival in dams with sterile intra-amniotic inflammation induced by the intra-amniotic injection of HMGB1.

### Clarithromycin prevents preterm labor and birth by interfering with the common pathway of parturition

**Fig. 2:**
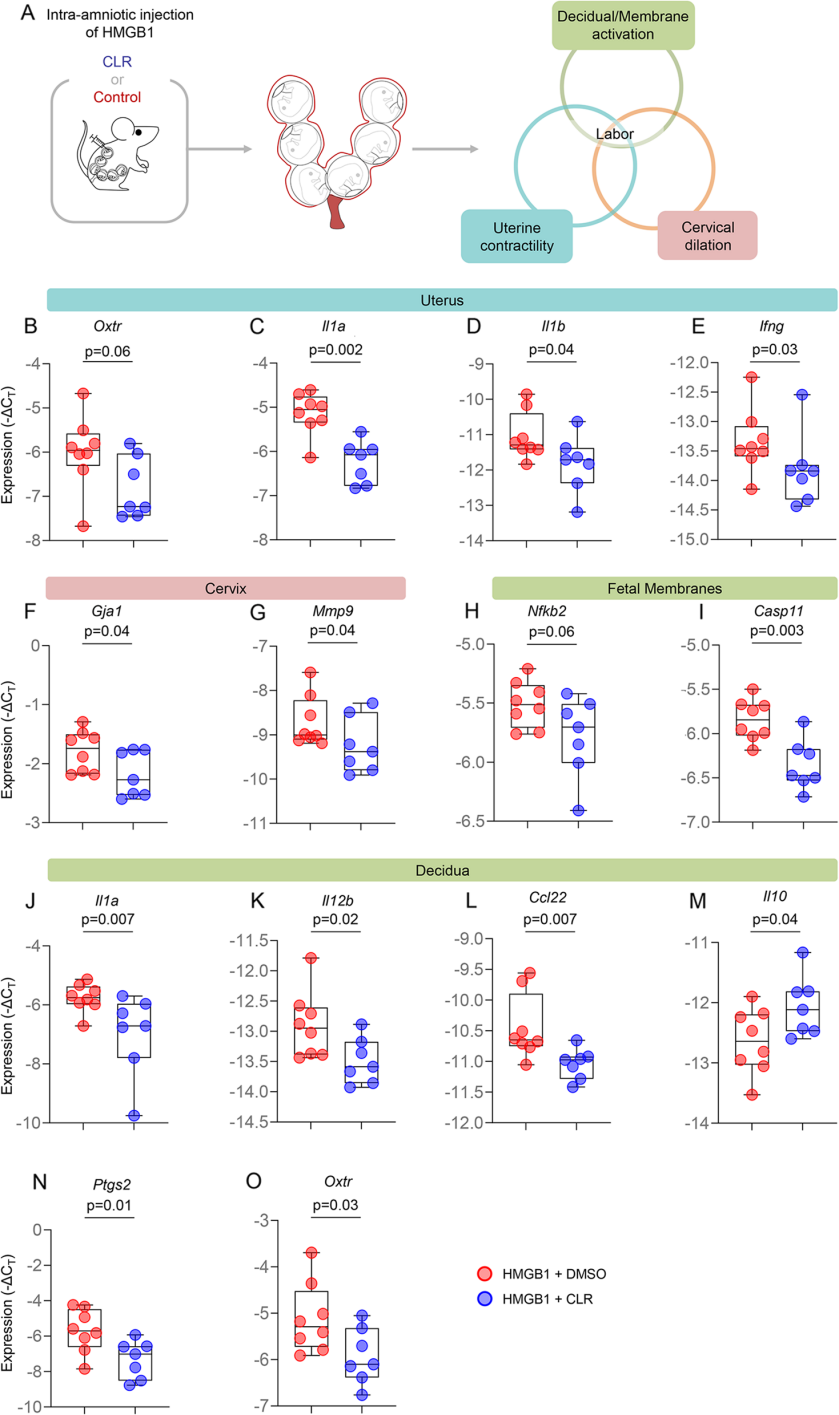
Clarithromycin interferes with the common pathway of labor. **A** Dams were intra-amniotically injected with HMGB1 under ultrasound guidance on 14.5 days *post coitum* (dpc) and treated with 75 mg/kg of clarithromycin (CLR; *n* = 7) or DMSO (vehicle control; *n* = 8) at 6, 12, 24, 48, 72, and 96 h post-injection. On 18.5 dpc, two hours after the last dose of CLR or DMSO, mice were euthanized and tissue collection was performed to obtain the decidua, uterus, cervix, fetal membranes, placenta, fetal lung, fetal intestine, fetal liver, and fetal spleen. Expression (-ΔC_T_) of **B** *Oxtr*, **C** *Il1a*, **D** *Il1b*, and **E** *Ifng* in the uteri of HMGB1-injected dams treated with CLR (blue dots) or DMSO (red dots). Expression (-ΔC_T_) of **F** *Gja1* and **G** *Mmp9* in the cervices of HMGB1-injected dams treated with CLR (blue dots) or DMSO (red dots). Expression (-ΔC_T_) of **H** *Nfkb2* and **I** *Casp11* in the fetal membranes of neonates born to HMGB1-injected dams treated with CLR (blue dots) or DMSO (red dots). Expression (-ΔC_T_) of **J** *Il1a*, **K** *Il12b*, **L** *Ccl22*, **M** *Il10*, **N** *Ptgs2*, and **O** *Oxtr* in the decidua of HMGB1-injected dams treated with CLR (blue dots) or DMSO (red dots). Data are represented as box-and-whisker plots with medians, interquartile ranges, and min/max ranges. *P*-values were determined using the one-sided Mann–Whitney U-test

Labor is a tightly regulated inflammatory process that requires the orchestrated activation of a common pathway including uterine contractility, cervical dilation, and decidual/fetal membrane activation [4, 65, 66]. Given that clarithromycin improved pregnancy outcomes in our model of HMGB1-induced preterm birth, we further investigated whether this beneficial effect was due to the effect of clarithromycin on the common pathway of labor (Fig. 2A). The uterine expression of *Oxtr*, a key regulator of uterine contraction [67], and the inflammatory genes *Il1a*, *Il1b*, and *Ifng* was reduced in dams intra-amniotically injected with HMGB1 and treated with clarithromycin compared to controls (Fig. 2B-E and Additional file 1). In the cervix, the expression of two crucial transcripts involved in cervical contraction and ripening, *Gja1* [68] and *Mmp9* [52, 69], respectively, was decreased upon treatment with clarithromycin (Fig. 2F&G and Additional file 1). Furthermore, the expression of the pro-inflammatory mediators *Nfkb2* and *Casp11* was decreased in the fetal membranes of dams injected with HMGB1 and treated with clarithromycin (Fig. 2H&I and Additional file 1). Decidual activation during labor involves the expression of multiple pro-inflammatory mediators [70–78], and we observed that treatment with clarithromycin had a potent anti-inflammatory effect in this tissue as indicated by the reduced expression of *Il1a*, *Il12b*, and *Ccl22* together with increased expression of the anti-inflammatory cytokine *Il10* (Fig. 2J-M and Additional file 1). Furthermore, the expression of the labor mediators *Ptgs2* [79–82] and *Oxtr* [67] was reduced in the decidua upon treatment with clarithromycin (Fig. 2N&O and Additional file 1). Together, these data show that clarithromycin exerts anti-inflammatory effects in the intrauterine tissues implicated in the common pathway of parturition, representing a potential mechanism whereby this antibiotic prevents alarmin-induced preterm birth.

### Clarithromycin does not reduce HMGB1-induced cytokine concentrations in the amniotic cavity

**Fig. 3:**
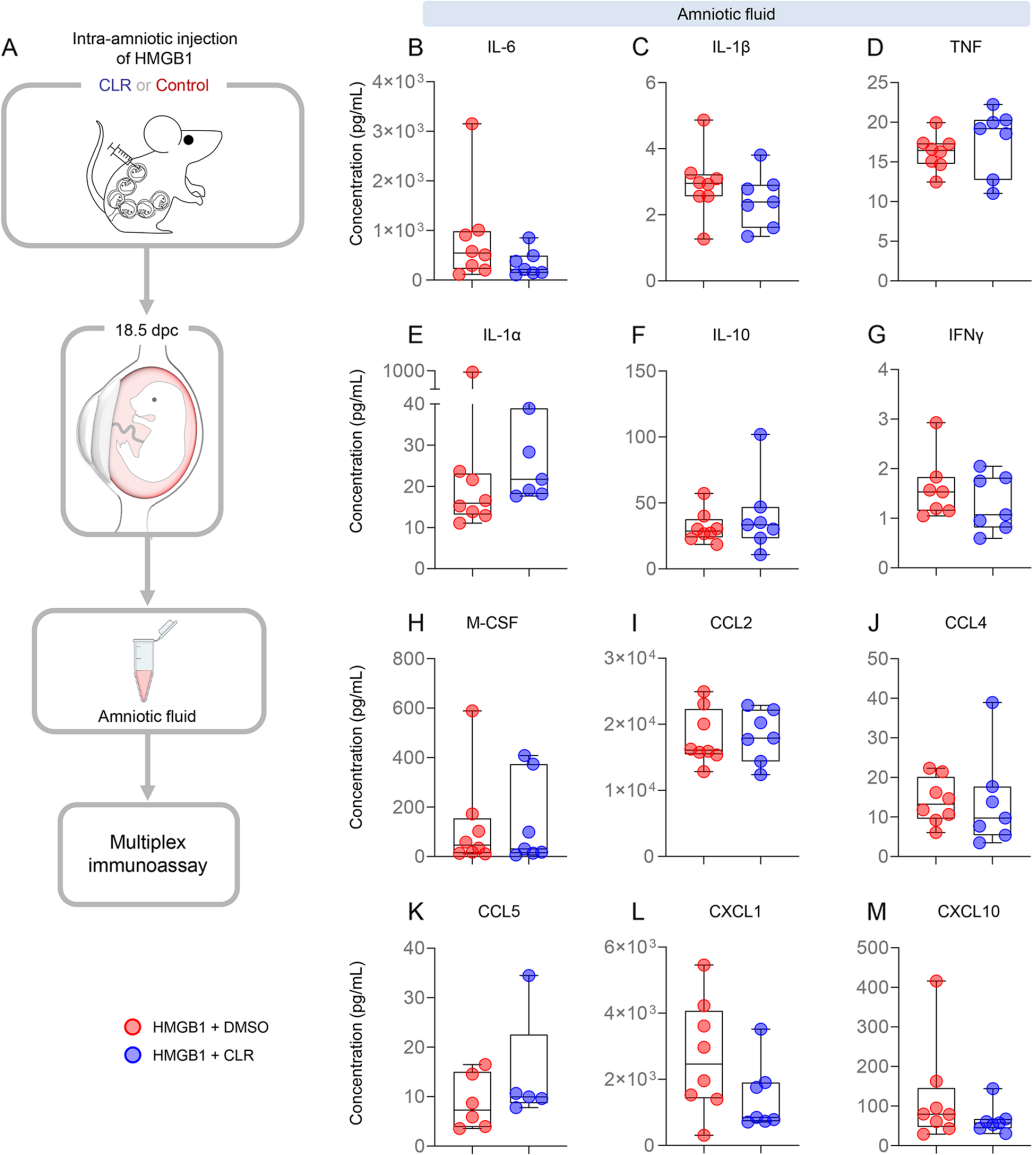
Clarithromycin does not alter HMGB1-induced cytokine concentrations in amniotic fluid. **A** Dams were intra-amniotically injected with HMGB1 under ultrasound guidance on 14.5 days *post coitum* (dpc) and treated with 75 mg/kg of clarithromycin (CLR; *n* = 7) or DMSO (vehicle control; *n* = 8) at 6, 12, 24, 48, 72, and 96 h post-injection. On 18.5 dpc, two hours after the last dose of CLR or DMSO, mice were euthanized and collection of amniotic fluid was performed to assess the concentrations of cytokines in the amniotic cavity. Amniotic fluid cytokine levels (pg/mL) of **B** IL-6, **C** IL-1β, **D** TNF, **E** IL-1α, **F** IL-10, **G** IFNγ, **H** M-CSF, **I** CCL2, **J** CCL4, **K** CCL5, **L** CXCL1, and **M** CXCL10 from dams intra-amniotically injected with HMGB1 and treated with CLR (blue dots) or DMSO (red dots). Data are represented as box-and-whisker plots with medians, interquartile ranges, and min/max ranges. *P*-values were determined using the one-sided Mann–Whitney U-test

Next, we evaluated whether treatment with clarithromycin improved adverse perinatal outcomes by reducing the inflammatory milieu induced by HMGB1 in the amniotic cavity. Amniotic fluid was collected from dams intra-amniotically injected with HMGB1 and treated with clarithromycin or vehicle control, and a multiplex immunoassay was performed to assess the concentrations of multiple cytokines (Fig. 3A). There were no significant differences in the amniotic fluid concentrations of all evaluated cytokines between dams receiving HMGB1 and treated with clarithromycin and those that received vehicle control (Fig. 3B-M). These data suggest that the improved neonatal outcomes induced by clarithromycin treatment are not solely explained by decreased concentrations of pro-inflammatory cytokines in the amniotic cavity.

### Clarithromycin has potent anti-inflammatory effects in the placenta

**Fig. 4:**
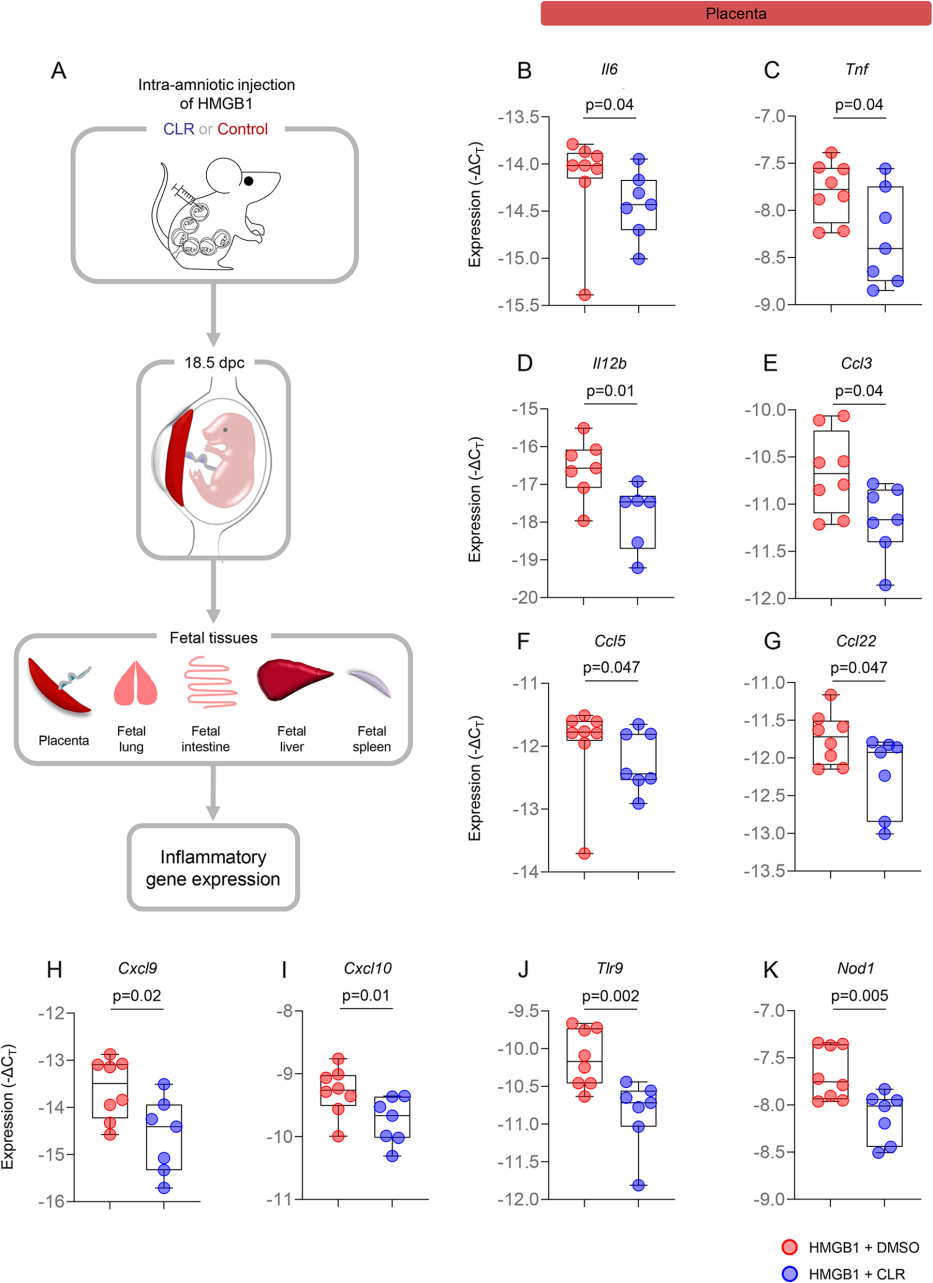
Clarithromycin downregulates inflammatory gene expression in the placenta. **A** Dams were intra-amniotically injected with HMGB1 under ultrasound guidance on 14.5 days *post coitum* (dpc) and treated with 75 mg/kg of clarithromycin (CLR; *n* = 7) or DMSO (vehicle control; *n* = 8) at 6, 12, 24, 48, 72, and 96 h post-injection. On 18.5 dpc, two hours after the last dose of CLR or DMSO, mice were euthanized and tissue collection was performed to obtain the placenta and assess inflammatory gene expression. Expression (-ΔC_T_) of **B** *Il6*, **C** *Tnf*, **D** *Il12b*, **E** *Ccl3*, **F** *Ccl5*, **G** *Ccl22*, **H** *Cxcl9*, **I** *Cxcl10*, **J** *Tlr9*, and **K** *Nod1* from dams intra-amniotically injected with HMGB1 and treated with CLR (blue dots) or DMSO (red dots). Data are represented as box-and-whisker plots with medians, interquartile ranges, and min/max ranges. *P*-values were determined using the one-sided Mann–Whitney U-test

Pregnant women with intra-amniotic inflammation are at increased risk of acute inflammatory lesions in the placenta [83]. Such lesions are associated with a high risk of both short- and long-term neonatal adverse outcomes including respiratory and intestinal pathologies [84–86]. Therefore, we sought to evaluate the effects of clarithromycin in the placentas of dams that received HMGB1. Placentas from dams intra-amniotically injected with HMGB1 and treated with clarithromycin or vehicle control were collected and the expression of inflammatory genes was evaluated (Fig. 4A). Importantly, the placental expression of *Il6* and *Tnf*, both of which play a critical role in the inflammatory process of parturition [61, 87–89], was downregulated by treatment with clarithromycin compared to control dams (Fig. 4B&C and Additional file 1). Moreover, multiple transcripts for inflammatory cytokines and chemokines, including *Il12b*, *Ccl3*, *Ccl5*, *Ccl22*, *Cxcl9*, *Cxcl10*, *Tlr9*, and *Nod1*, were also downregulated in the placentas of dams that received intra-amniotic HMGB1 and were treated with clarithromycin (Fig. 4D-K and Additional file 1). These data suggest that clarithromycin reduces the placental inflammation induced by intra-amniotic HMGB1, providing a potential mechanism whereby this macrolide improves neonatal survival.

### Clarithromycin ameliorates inflammation in the fetal tissues

**Fig. 5:**
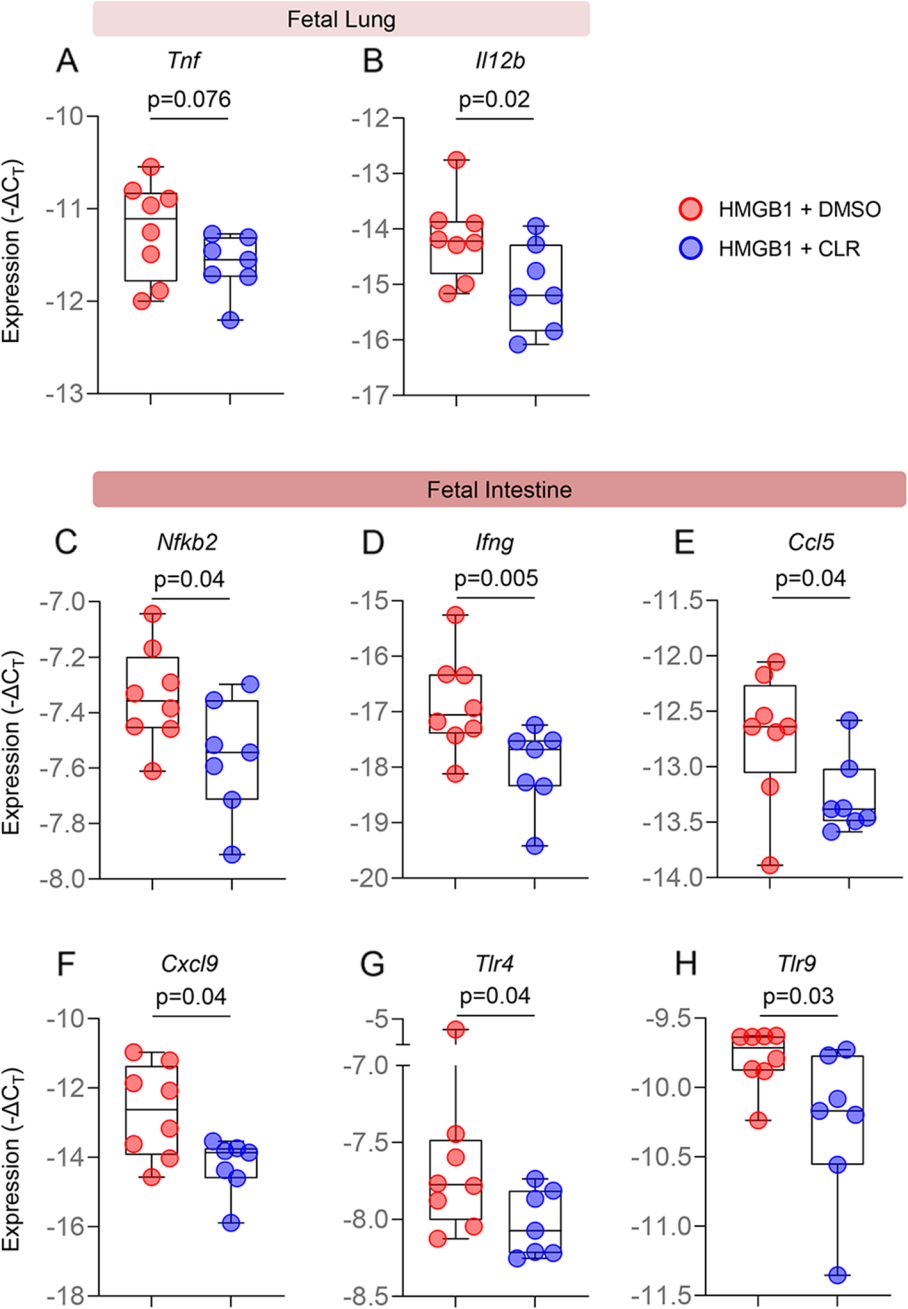
Clarithromycin downregulates inflammatory gene expression in the fetal lung and intestine. Expression (-ΔC_T_) of **A** *Tnf* and **B** *Il12b* in the lungs of fetuses from dams intra-amniotically injected with HMGB1 and treated with CLR (blue dots) or DMSO (red dots). Expression (-ΔC_T_) of **C** *Nfkb2*, **D** *Ifng*, **E** *Ccl5*, **F** *Cxcl9*, **G** *Tlr4*, and **H** *Tlr9* in the intestines of fetuses from dams intra-amniotically injected with HMGB1 and treated with CLR (blue dots) or DMSO (red dots). Data are represented as box-and-whisker plots with medians, interquartile ranges, and min/max ranges. *P*-values were determined using the one-sided Mann–Whitney U-test

The most common pathology in preterm neonates is respiratory distress syndrome [90, 91], while one of the most devastating pathologies associated with prematurity is necrotizing enterocolitis [92]. Thus, we next investigated the effects of maternal clarithromycin treatment in the lung and intestine of fetuses exposed to intra-amniotic HMGB1. The lung and intestine of fetuses from dams injected with HMGB1 and treated with clarithromycin or vehicle control were collected prior to delivery to evaluate the expression of inflammatory mediators (Fig. 4A). We found that the expression of *Tnf* and *Il12b* was downregulated in the fetal lung from dams exposed to HMGB1 and treated with clarithromycin compared to those born to vehicle control dams (Fig. 5A&B and Additional file 1). Moreover, clarithromycin treatment downregulated the expression of multiple inflammatory mediators such as *Nfkb2*, *Ifng*, *Ccl5*, *Cxcl9*, *Tlr4*, and *Tlr9* in the intestine of fetuses exposed to HMGB1 (Fig. 5C-H and Additional file 1).

**Fig. 6:**
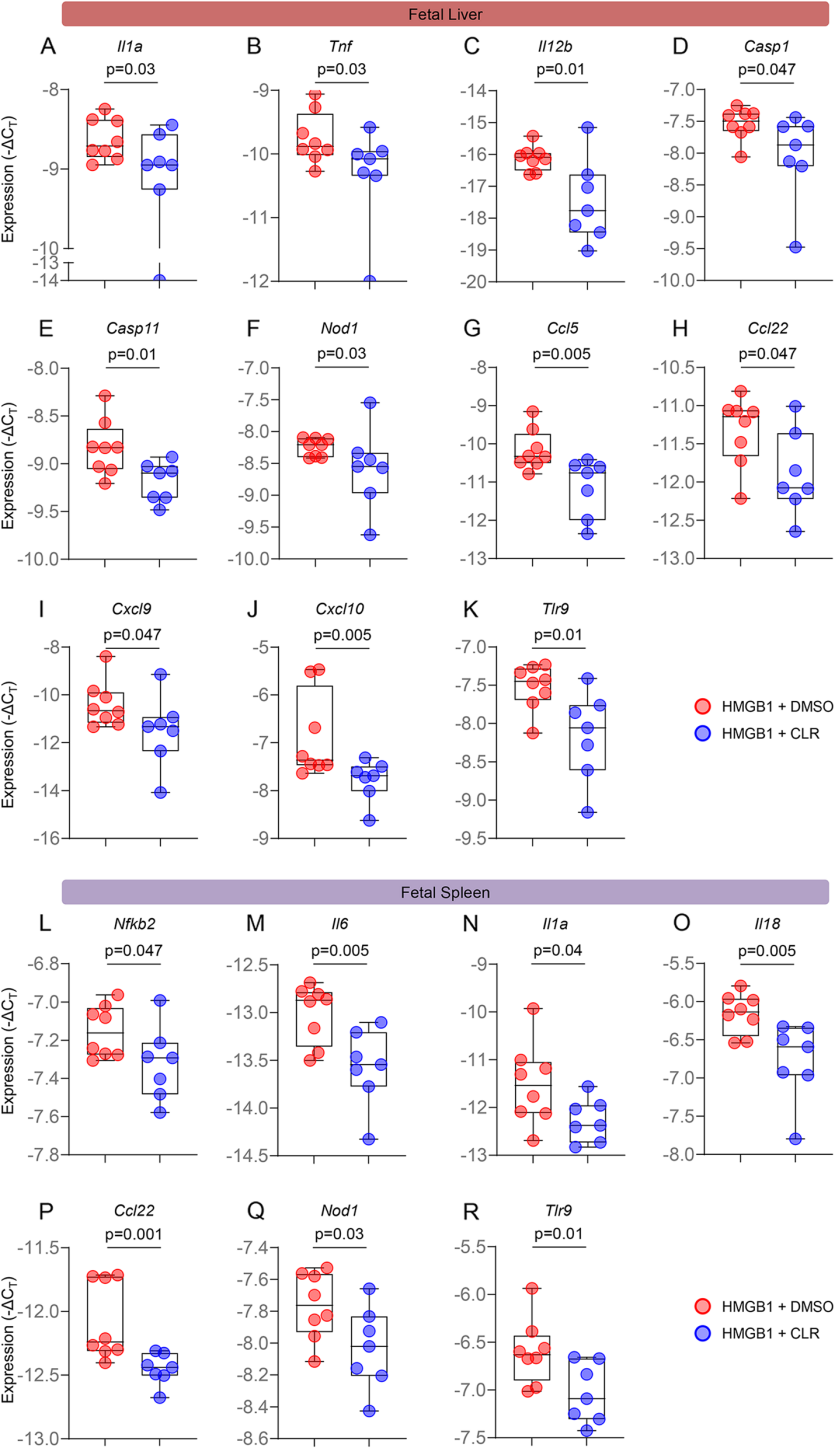
Clarithromycin downregulates inflammatory gene expression in the fetal liver and spleen. Expression (-ΔC_T_) of **A** *Il1a*, **B** *Tnf*, **C** *Il12b*, **D** *Casp1*, **E** *Casp11*, **F** *Nod1*, **G** *Ccl5*, **H** *Ccl22*, **I** *Cxcl9*, **J** *Cxcl10*, and **K** *Tlr9* in the livers of fetuses from dams intra-amniotically injected with HMGB1 and treated with CLR (blue dots) or DMSO (red dots). Expression (-ΔC_T_) of **L** *Nfkb2*, **M** *Il6*, **N** *Il1a*, **O** *Il18*, **P** *Ccl22*, **Q** *Nod1*, and **R** *Tlr9* in the spleens of fetuses from dams intra-amniotically injected with HMGB1 and treated with CLR (blue dots) or DMSO (red dots). Data are represented as box-and-whisker plots with medians, interquartile ranges, and min/max ranges. *P*-values were determined using the one-sided Mann–Whitney U-test

The liver is the most important metabolic organ [93]. During fetal life, the liver plays an important role in hematopoiesis and protein synthesis [94, 95]. On the other hand, the spleen is the largest secondary lymphatic organ [96] and is critical for fetal adaptive immunity [97, 98]. Therefore, we evaluated whether maternal clarithromycin treatment modulates the expression of inflammatory mediators in such fetal organs. The liver and spleen were also collected prior to delivery from fetuses of dams intra-amniotically injected with HMGB1 that received clarithromycin or vehicle control (Fig. 4A). The fetal liver from dams treated with clarithromycin displayed downregulated expression of multiple pro-inflammatory immune mediators such as *Il1a*, *Tnf*, *Il12b*, *Casp1*, *Casp11*, *Nod1*, *Ccl5*, *Ccl22*, *Cxcl9*, *Cxcl10*, and *Tlr9* compared to that of fetuses born to vehicle control dams (Fig. 6A-K and Additional file 1). Similarly, the expression of multiple inflammatory mediators, including *Nfkb2*, *Il6*, *Il1a*, *Il18*, *Ccl22*, *Nod1*, and *Tlr9*, was downregulated in the fetal spleen from dams that were exposed to HMGB1 and received clarithromycin (Fig. 6L-R and Additional file 1).

Collectively, these findings indicate that clarithromycin acts as a modulator of the inflammatory response in fetuses exposed to HMGB1, which may contribute to the improved neonatal outcomes observed after treatment with this antibiotic.

## Discussion

One of every four premature neonates is born to a woman with intra-amniotic inflammation [27, 99, 100], which was largely attributed to cultivable and non-cultivable microbes invading the amniotic cavity (i.e., infection) [22, 25–29]. Therefore, much research has been focused on exploring the appropriate antibiotic regimen for treating intra-amniotic infection [45–49]. Such efforts have been fruitful by demonstrating that the correct antibiotics (including clarithromycin) can eradicate intra-amniotic infection [45–49, 101], reduce intra-amniotic inflammation [45–49], and, more importantly, extend gestational length and prevent adverse neonatal outcomes, including intra-ventricular hemorrhage, peri-ventricular leukomalacia, and cerebral palsy [102]. Yet, recent research has provided solid evidence that intra-amniotic inflammation can occur in the absence of detectable and cultivable bacteria (i.e., sterile intra-amniotic inflammation) [26–29]. In such a setting, we have proposed that the optimal treatment strategy includes the utilization of drugs with anti-inflammatory properties [14, 17, 103], including antibiotics [45–48]. Indeed, a recent report showed that the macrolide clarithromycin can be used to treat women with sterile intra-amniotic inflammation who were destined to deliver preterm [48]. Herein, we put forth mechanistic evidence showing that clarithromycin can be utilized to prevent preterm birth and adverse neonatal outcomes in an animal model of sterile intra-amniotic inflammation.

In the current study, we explored the mechanisms whereby clarithromycin prevents preterm birth and adverse neonatal outcomes. First, we report that this macrolide interferes with the common pathway of labor by reducing the gene expression of contractility-associated proteins such as OXTR, CX43, and COX2, which are increased in preterm and term labor [104]. This finding is in line with previous in vitro demonstrations showing that clarithromycin inhibits oxytocin-induced myometrial contractility [105]. Furthermore, we have previously shown that treatment with clarithromycin prevented the premature onset of labor induced by *Ureaplasma parvum* [15]. Notably, in the current study, we showed that treatment with clarithromycin reduced the expression of several inflammatory mediators in the intra-uterine tissues (e.g., uterus, fetal membranes, decidua, and placenta) involved in the cascade of parturition. These data are consistent with previous demonstrations in non-reproductive tissues that clarithromycin suppresses the production and secretion of inflammatory cytokines by interfering with the AP-1 and NF-κB pathways [106, 107]. Specifically, clarithromycin suppressed NF-κB-mediated pro-inflammatory cytokine production by interfering with the mitochondrial proteins 4-nitrophenylphosphatase domain and non-neuronal synaptosomal associated protein 25-like protein homolog (NIP-SNAP)-1 and -2 [107]. Furthermore, clarithromycin can dampen inflammation by modulating the concentrations of chemokines such as IL-8 [106], which results in reduced neutrophil infiltration at the site of injury [108]. Alternatively, clarithromycin can foster an anti-inflammatory milieu by increasing suppressive cytokines such as IL-10 [109], as shown in the decidua herein, and by expanding immunosuppressive innate immune cells [e.g., myeloid-derived suppressor cell (MDSC)-like cells] [110]. Taken together, these data indicate that clarithromycin prevents preterm birth largely by interfering with the inflammatory cascade of labor in the intra-uterine maternal tissues (uterine decidua and cervix).

The strategy of using anti-inflammatory approaches to tackle prematurity has been utilized by us and others [111–118]. For example, we have shown that anti-inflammatory drugs and peptides such as MCC950 (an NLRP3 inflammasome inhibitor [119]), rosiglitazone (an anti-diabetic thiazolidinedione drug [120]), and exendin-4 [an agonist of the glucagon-like protein-1 receptor (GLP1R) [121]] prevent preterm birth induced by inflammation in mice [12–14, 53–55, 122]. Yet, such strategies are not approved for use in pregnant women. To address this need, we and others have also utilized progesterone and betamethasone, drugs approved for pregnant women, to prevent inflammation-induced preterm birth in mice [17, 52, 103, 123, 124]. Notably, betamethasone can prevent preterm birth in the animal model of sterile intra-amniotic inflammation used herein [17]. Pertinent to the latter finding, clarithromycin has been shown to be as potent as corticosteroids (e.g., prednisolone) in inhibiting the production of pro-inflammatory cytokines [125]. Therefore, the use of clarithromycin to treat inflammation-induced preterm birth is well-supported.

In the current study, we showed that treatment with clarithromycin reduced the expression of several inflammatory mediators in the placenta. This finding could be explained by previous pharmacokinetics studies showing that clarithromycin efficiently crosses the human placenta [50, 51]. Indeed, clarithromycin is more efficient than other macrolides, such as azithromycin and erythromycin, at crossing the placenta [50]. The mechanisms whereby clarithromycin reduces placental inflammation must involve the inhibition of the NF-κB pathway, as has been demonstrated in other systems [106, 107] and the uterine decidua herein. Furthermore, clinical studies reported that the placentas of patients treated with clarithromycin displayed a lesser degree of histological funisitis (acute inflammation of the umbilical cord) [102, 126]. However, we also found that treatment with clarithromycin did not reduce inflammation in the amniotic cavity, as was observed in humans [45–48]; yet, a direct comparison between animal and human studies requires careful consideration. Regardless, it is tempting to suggest that the reason why clarithromycin did not fully prevent neonatal mortality induced by HMGB1 lies within its minimal effects in the amniotic cavity in our model. In the current study, another point to consider is that we utilized concentrations of clarithromycin similar to those utilized in humans [57, 58], given that macrolides can exhibit cytotoxicity at high concentrations [127]. Together, these findings also allow us to propose that the combination of approved approaches (e.g., betamethasone and clarithromycin) may be more effective for reducing the intra-amniotic inflammatory milieu compared to clarithromycin alone. Nevertheless, further research is required to test such a proposal.

A major finding of our study is that treatment with clarithromycin dampened inflammation in the fetal tissues, namely the lung, intestine, and liver. To our knowledge, this is the first evidence showing the anti-inflammatory effects of clarithromycin in a model of sterile intra-amniotic inflammation. Similarly, previous mechanistic studies in animal models have consistently shown that macrolides, such as azithromycin, reduce *Ureaplasma-*induced inflammatory markers in the amniotic cavity, fetal lung, and/or fetal skin [128–130]. Indeed, such a decrease in the local inflammatory response has been translated to improvements in physiological parameters, such as fetal cardiac output [130]. Furthermore, clarithromycin has been successfully used to diminish inflammation in patients with lung pathologies (e.g., asthma [108, 131], bronchiectasis [132], and cystic fibrosis [133]). The protective effects of clarithromycin have also been reported in gastrointestinal diseases (e.g., *Helicobacter pylori* infection [134], intestinal mucositis [135], and Crohn's disease [136]). In line with the abovementioned studies, we also report that clarithromycin dampens inflammation in the fetal spleen, whose inflammatory status can serve as a marker (Doppler of the fetal splenic vein) of fetal damage induced by intra-amniotic inflammation [137]. Collectively, these data suggest that the mechanisms whereby clarithromycin improves neonatal survival include dampening of inflammation in the fetal organs. However, further mechanistic demonstrations are required to investigate whether neonates born to women treated with clarithromycin are immunocompetent.

The current study has some limitations. There is a lack of information regarding the inflammatory effects induced by HMGB1 alone in gestational tissues utilizing in vivo models. Ongoing research in our lab is investigating such effects; yet, we have previously shown that the in vitro treatment of the chorioamniotic membranes with HMGB1 drives a similar inflammatory response [42] to that observed in the current study. Thus, we surmise that the vehicle control group (HMGB1 + DMSO) would have similar results in mice injected by HMGB1 alone. Another limitation is that, for the mice utilized for tissue collection, we could not distinguish between those mice that would have delivered preterm from those that would have delivered at term. Ongoing research in our lab is also focused on establishing non-invasive approaches to monitor the progression of labor in mice and identify those that can benefit from treatments to prevent preterm birth.

## Conclusions

In summary, this study provides evidence that clarithromycin can be utilized to prevent preterm birth and improve neonatal survival in the context of sterile intra-amniotic inflammation. The mechanisms whereby clarithromycin prevents preterm birth involve interference with the common cascade of parturition, which is largely governed by the uterine and cervical tissues. Furthermore, clarithromycin had strong anti-inflammatory effects in the fetal tissues, providing a possible role for this macrolide in dampening fetal inflammatory responses, which translates to improved neonatal outcomes. Collectively, these data indicate that clarithromycin can be used in cases of sterile intra-amniotic inflammation, a condition that currently lacks treatment.

## Supplementary Information

**Additional file 1.** RT-qPCR gene expression data. -ΔC_T_ values for gene expression in the maternal and fetal tissues from mice treated with clarithromycin (CLR) or DMSO (vehicle control) reported in this study.

**Additional file 2:** **Table S1. **List of TaqMan® gene expression assays utilized for RT-qPCR. 

## Data Availability

All of the data generated or analyzed during this study are included in this published article or its supporting information. Gene expression data are provided as Additional File 1.

## Abbreviations

DAMP: Damage-associated molecular pattern
DMSO: Dimethyl sulfoxide
GLP1R: Glucagon-like protein-1 receptor
HMGB1: High-mobility group box-1
HSP70: Heat-shock protein-70
IACUC: Institutional Animal Care and Use Committee
MDSC: Myeloid-derived suppressor cell
NLRP3: NLR family pyrin domain-containing-3
S100B: S100 calcium binding protein-B
