# Supplementary material for: Clarithromycin prevents preterm birth and neonatal mortality by dampening alarmin-induced maternal–fetal inflammation in mice
Source: BMC Pregnancy Childbirth. 2022 Jun 20;22:503. doi: 10.1186/s12884-022-04764-2 (PMC9210693; doi:10.1186/s12884-022-04764-2)
Supplement: Supplementary file 2 — Additional file 2: Table S1. List of TaqMan® gene expression assays utilized for RT-qPCR. [file 12884_2022_4764_MOESM2_ESM.docx]

| **Additional file 2 Table S1.** List of TaqMan® gene expression assays utilized for the RT-qPCR. **Gene Name** | **Symbol** | **Assay ID** |
| --- | --- | --- |
| Caspase 1 | *Casp1* | Mm00438023_m1 |
| SR-related CTD-associated factor 11 | *Scaf11 (Casp11)* | Mm01297328_m1 |
| Chemokine (C-C motif) ligand 3 | *Ccl3* | Mm00441259_g1 |
| Chemokine (C-C motif) ligand 5 | *Ccl5* | Mm01302427_m1 |
| Chemokine (C-C motif) ligand 22 | *Ccl22* | Mm00436439_m1 |
| Chemokine (C-X-C motif) ligand 9 | *Cxcl9* | Mm00434946_m1 |
| Chemokine (C-X-C motif) ligand 10 | *Cxcl10* | Mm00445235_m1 |
| Gap junction protein, alpha 1 | *Gja1* | Mm00439105_m1 |
| Interferon gamma | *Ifng* | Mm01168134_m1 |
| Interleukin 1 alpha | *Il1a* | Mm00439620_m1 |
| Interleukin 1 beta | *Il1b* | Mm00434228_m1 |
| Interleukin 6 | *Il6* | Mm00446190_m1 |
| Interleukin 10 | *Il10* | Mm01288386_m1 |
| Interleukin 12b | *Il12b* | Mm01288989_m1 |
| Interleukin 18 | *Il18* | Mm00434226_m1 |
| Matrix metallopeptidase 9 | *Mmp9* | Mm00442991_m1 |
| Nuclear factor of kappa light polypeptide gene enhancer in B cells 2, p49/p100 | *Nfkb2* | Mm00479807_m1 |
| Nucleotide-binding oligomerization domain containing 1 | *Nod1* | Mm00805062_m1 |
| Oxytocin receptor | *Oxtr* | Mm01182684_m1 |
| Prostaglandin-endoperoxide synthase 2 | *Ptgs2* | Mm00478374_m1 |
| Toll-like receptor 4 | *Tlr4* | Mm00445273_m1 |
| Toll-like receptor 9 | *Tlr9* | Mm00446193_m1 |
| Tumor necrosis factor | *Tnf* | Mm00443258_m1 |
| Actin, beta | *Actb* | Mm04394036_g1 |
| Glyceraldehyde-3-phosphate dehydrogenase | *Gapdh* | Mm99999915_g1 |
| Glucuronidase, beta | *Gusb* | Mm01197698_m1 |
| Heat shock protein 90 alpha (cytosolic), class B member 1 | *Hsp90ab1* | Mm00833431_g1 |
